# Genome Sequences of Bacteriophages ClearAsMud and Kauala, Isolated from Microbacterium foliorum

**DOI:** 10.1128/MRA.01026-20

**Published:** 2020-11-12

**Authors:** Heidi Lin, Michael Reeves, Melissa Acevedo, Kelsey Bass, Elizabeth Chau, Brandon Ching, Elisaelena Enriquez, Skylar Evans, Kaitlyn Mamora, Caitlyn Pang, Michelle Santos, Chelsea Tafoya, Madyllyne Vaca, Wiliam Van Iderstein, Luis Velasco, Vivianna Williams, Grant Yonemoto, Tyler Yonemoto, Jessica Choi, Natasha Dean, Arturo Diaz

**Affiliations:** a Biology Department, La Sierra University, Riverside, California, USA; Queens College

## Abstract

Cluster EC ClearAsMud and cluster EA4 Kauala are lytic *Siphoviridae* bacteriophages that were isolated from soil in southern California using Microbacterium foliorum NRRL B-24224 as the host. The ClearAsMud and Kauala genomes are 52,987 bp and 39,378 bp, respectively, and contain 92 and 56 predicted protein-coding genes, respectively.

## ANNOUNCEMENT

**FIG 1:**
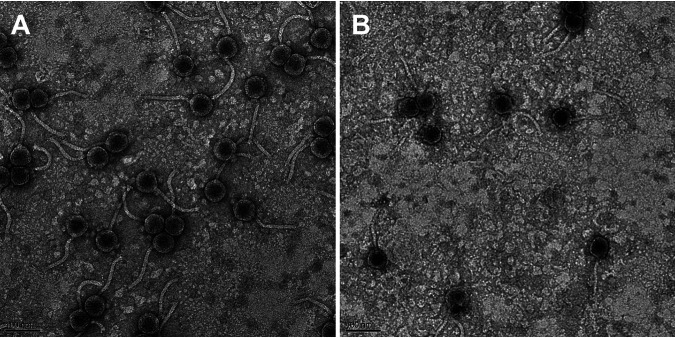
Transmission electron micrographs of *Microbacterium* phages ClearAsMud (A) and Kauala (B). Phage lysates were negatively stained with 1% uranyl acetate.

In order to characterize viral diversity and evolution, ClearAsMud and Kauala were isolated as part of the La Sierra University 2020 Science Education Alliance-Phage Hunters Advancing Genomics and Evolutionary Science (SEA-PHAGES) course (1). ClearAsMud was isolated from a mud sample from Riverside, California, while Kauala was isolated from a potted plant in Rancho Cucamonga, California. Phages were purified and amplified in Microbacterium foliorum NRRL B-24224 grown in peptone-yeast-calcium agar (PYCa) medium at 30°C, with shaking at 250 rpm. Both phages produced clear plaques and lacked integrase genes. Negative-staining transmission electron microscopy showed that both phages had icosahedral capsids with flexible, noncontractile tails (Fig. 1).

After two rounds of plaque purification, DNA was purified from high-titer lysates using the Wizard DNA clean-up kit (A7280; Promega). Sequencing libraries were prepared using the NEBNext Ultra II FS DNA library prep kit with dual-indexed barcoding. The libraries were run on an Illumina MiSeq instrument, yielding 2.2 million and 925,630 paired-end 150-base reads for Kauala and ClearAsMud, respectively. This was sufficient to provide 27-fold and 7,821-fold coverage for ClearAsMud and Kauala, respectively. Raw reads were assembled with Newbler (v2.9) with default settings, yielding a single phage contig for each, and results were checked for completeness, accuracy, and genome termini using Consed. The genomes were manually annotated using the Phage Evidence Collection and Annotation Network (PECAAN) (http://pecaan.kbrinsgd.org), which collects information from Starterator (v1.2), Glimmer (v3.02) (2), and GeneMark (v2.5) (3) to identify start sites and coding capacity and from PhagesDB with BLAST (4), HHpred (v3.2) (5), NCBI BLAST (6), the Conserved Domain Database (7), TMHMM (v2.0) (8), TOPCONS (v2.0) (9), and tRNAscan-SE (v2.0) (10) for evidence of gene function. Default parameters were used for all software unless otherwise specified.

ClearAsMud and Kauala were placed in cluster EC and subcluster EA4, respectively, based on criteria described previously (11, 12). Both have circularly permuted genome ends, as the genomes had different starting and ending locations and were slightly longer than one genome length (13). The ClearAsMud genome is 52,987 bp long, with a G+C content of 69%. Putative functions were assigned for 37 of the 92 predicted protein-coding genes. As is the case for all cluster EC phages, all of the ClearAsMud open reading frames are transcribed in the forward direction. Characteristic of cluster EC phages, ClearAsMud contains 9 copies of a unique 14-bp sequence (CTATAGGTGTAAGC) found 19 to 30 bp upstream of transcriptional start sites. Interestingly, ClearAsMud and KaiHaiDragon, another cluster EC phage discovered on the campus of La Sierra University, are 91.5% identical based on average nucleotide identity (ANI) as determined by OrthoANI (14).

The Kauala genome has a G+C content of 64.4% and is 39,378 bp long. Of a total of 56 genes, 26 genes were assigned a function; no tRNA was identified. Kauala showed genomewide nucleotide similarity to other phages in its subcluster, with 90.27% and 90.26% identities to its closest relatives Sinatra (GenBank accession number MK937602) and PrincePhergus (GenBank accession number MK620901), respectively. Additional details on ClearAsMud and Kauala can be found in the Actinobacteriophage Database (https://phagesdb.org/phages/ClearAsMud/ for ClearAsMud and https://phagesdb.org/phages/Kauala/ for Kauala) (4).

### Data availability.

GenBank and SRA accession numbers are MT657336 and SRX8516562, respectively, for ClearAsMud and MT657344 and SRX8516563, respectively, for Kauala.

## References

[1] Jordan TC, Burnett SH, Carson S, Caruso SM, Clase K, DeJong RJ, Dennehy JJ, Denver DR, Dunbar D, Elgin SCR, Findley AM, Gissendanner CR, Golebiewska UP, Guild N, Hartzog GA, Grillo WH, Hollowell GP, Hughes LE, Johnson A, King RA, Lewis LO, Li W, Rosenzweig F, Rubin MR, Saha MS, Sandoz J, Shaffer CD, Taylor B, Temple L, Vazquez E, Ware VC, Barker LP, Bradley KW, Jacobs-Sera D, Pope WH, Russell DA, Cresawn SG, Lopatto D, Bailey CP, Hatfull GF 2014 A broadly implementable research course in phage discovery and genomics for first-year undergraduate students. mBio 5:e01051-13. doi:10.1128/mBio.01051-13. 24496795 PMC3950523

[2] Delcher AL, Bratke KA, Powers EC, Salzberg SL 2007 Identifying bacterial genes and endosymbiont DNA with Glimmer. Bioinformatics 23:673–679. doi:10.1093/bioinformatics/btm009. 17237039 PMC2387122

[3] Besemer J, Borodovsky M 2005 GeneMark: Web software for gene finding in prokaryotes, eukaryotes, and viruses. Nucleic Acids Res 33:451–454. doi:10.1093/nar/gki487. 15980510 PMC1160247

[4] Russell DA, Hatfull GF 2017 PhagesDB: the Actinobacteriophage Database. Bioinformatics 33:784–786. doi:10.1093/bioinformatics/btw711. 28365761 PMC5860397

[5] Söding J, Biegert A, Lupas AN 2005 The HHpred interactive server for protein homology detection and structure prediction. Nucleic Acids Res 33:W244–W248. doi:10.1093/nar/gki408. 15980461 PMC1160169

[6] Altschul SF, Gish W, Miller W, Myers EW, Lipman DJ 1990 Basic local alignment search tool. J Mol Biol 215:403–410. doi:10.1016/S0022-2836(05)80360-2. 2231712

[7] Marchler-Bauer A, Derbyshire MK, Gonzales NR, Lu S, Chitsaz F, Geer LY, Geer RC, He J, Gwadz M, Hurwitz DI, Lanczycki CJ, Lu F, Marchler GH, Song JS, Thanki N, Wang Z, Yamashita RA, Zhang D, Zheng C, Bryant SH 2015 CDD: NCBI's conserved domain database. Nucleic Acids Res 43:D222–D226. doi:10.1093/nar/gku1221. 25414356 PMC4383992

[8] Krogh A, Larsson B, von Heijne G, Sonnhammer ELL 2001 Predicting transmembrane protein topology with a Markov model: application to complete genomes. J Mol Biol 305:567–580. doi:10.1006/jmbi.2000.4315. 11152613

[9] Tsirigos KD, Peters C, Shu N, Kall L, Elofsson A 2015 The TOPCONS web server for consensus prediction of membrane protein topology and signal peptides. Nucleic Acids Res 43:W401–W407. doi:10.1093/nar/gkv485. 25969446 PMC4489233

[10] Lowe T, Chan P 2016 tRNAscan-SE On-line: integrating search and context for analysis of transfer RNA genes. Nucleic Acids Res 44:W54–W57. doi:10.1093/nar/gkw413. 27174935 PMC4987944

[11] Hatfull GF, Jacobs-Sera D, Lawrence JG, Pope WH, Russell DA, Ko C-C, Weber RJ, Patel MC, Germane KL, Edgar RH, Hoyte NN, Bowman CA, Tantoco AT, Paladin EC, Myers MS, Smith AL, Grace MS, Pham TT, O'Brien MB, Vogelsberger AM, Hryckowian AJ, Wynalek JL, Donis-Keller H, Bogel MW, Peebles CL, Cresawn SG, Hendrix RW 2010 Comparative genomic analysis of 60 mycobacteriophage genomes: genome clustering, gene acquisition, and gene size. J Mol Biol 397:119–143. doi:10.1016/j.jmb.2010.01.011. 20064525 PMC2830324

[12] Cresawn SG, Bogel M, Day N, Jacobs-Sera D, Hendrix RW, Hatfull GF 2011 Phamerator: a bioinformatics tool for comparative bacteriophage genomics. BMC Bioinformatics 12:395. doi:10.1186/1471-2105-12-395. 21991981 PMC3233612

[13] Merrill BD, Ward AT, Grose JH, Hope S 2016 Software-based analysis of bacteriophage genomes, physical ends, and packaging strategies. BMC Genomics 17:679. doi:10.1186/s12864-016-3018-2. 27561606 PMC5000459

[14] Lee I, Ouk Kim Y, Park S-C, Chun J 2016 OrthoANI: an improved algorithm and software for calculating average nucleotide identity. Int J Syst Evol Microbiol 66:1100–1103. doi:10.1099/ijsem.0.000760. 26585518

